# Combined measures of mimetic fidelity explain imperfect mimicry in a brood parasite–host system

**DOI:** 10.1098/rsbl.2022.0538

**Published:** 2023-02-15

**Authors:** Tanmay Dixit, Gary P. T. Choi, Salem al-Mosleh, Jess Lund, Jolyon Troscianko, Collins Moya, L. Mahadevan, Claire N. Spottiswoode

**Affiliations:** ^1^ Department of Zoology, University of Cambridge, Cambridge, UK; ^2^ Department of Mathematics, Massachusetts Institute of Technology, Cambridge, MA, USA; ^3^ School of Engineering and Applied Sciences, Harvard University, Cambridge, MA, USA; ^4^ Department of Physics, Harvard University, Cambridge, MA, USA; ^5^ Department of Organismic and Evolutionary Biology, Harvard University, Cambridge, MA, USA; ^6^ Centre for Ecology and Conservation, University of Exeter, Penryn, UK; ^7^ Musumanene Farm, Choma, Zambia; ^8^ DST-NRF Centre of Excellence at the FitzPatrick Institute of African Ornithology, University of Cape Town, Rondebosch, Cape Town, South Africa

**Keywords:** coevolution, avian brood parasitism, imperfect mimicry, mimetic fidelity, receiver perception, Minkowski functionals

## Abstract

The persistence of imperfect mimicry in nature presents a challenge to mimicry theory. Some hypotheses for the existence of imperfect mimicry make differing predictions depending on how mimetic fidelity is measured. Here, we measure mimetic fidelity in a brood parasite–host system using both trait-based and response-based measures of mimetic fidelity. Cuckoo finches *Anomalospiza imberbis* lay imperfectly mimetic eggs that lack the fine scribbling characteristic of eggs of the tawny-flanked prinia *Prinia subflava*, a common host species. A trait-based discriminant analysis based on Minkowski functionals—that use geometric and topological morphometric methods related to egg pattern shape and coverage—reflects this consistent difference between host and parasite eggs. These methods could be applied to quantify other phenotypes including stripes and waved patterns. Furthermore, by painting scribbles onto cuckoo finch eggs and testing their rate of rejection compared to control eggs (i.e. a response-based approach to quantify mimetic fidelity), we show that prinias do not discriminate between eggs based on the absence of scribbles. Overall, our results support relaxed selection on cuckoo finches to mimic scribbles, since prinias do not respond differently to eggs with and without scribbles, despite the existence of this consistent trait difference.

## 1. Introduction

Although mimics evolve to resemble models, imperfect mimicry is common in nature [1,2]. Explanations proposed for the persistence of imperfect mimicry (reviewed in [1–4]) include relaxed selection, competing selection pressures, and the evolution of models away from mimics. To test the predictive power of these explanations, we must quantify imperfect mimicry in biological systems.

Two classes of methods can quantify imperfect mimicry [2,5]. Trait-based approaches quantify trait differences between models and mimics. Response-based measures quantify differences in receiver response to models and mimics. Different hypotheses for imperfect mimicry entail different expectations for trait and response differences. For instance, the hypothesis that there is relaxed selection for perfect mimicry predicts that trait differences exist, but that receiver responses to models and mimics should be similar [2]. The hypothesis that models evolve away from mimics predicts the existence of both trait differences and differences in receiver responses to models and mimics [2]. Therefore, considering both trait-based and response-based measures of mimetic fidelity is crucial to determine causes of imperfect mimicry. However, very few studies have explicitly quantified both measures for a mimetic phenotype [5].

Here, we quantify trait-based and response-based measures of imperfect mimicry in an avian brood parasite–host system. Interspecific brood parasites, such as our study species, the cuckoo finch, lay eggs in the nests of other species (hosts) such as the tawny-flanked prinia, passing costs of parenthood onto hosts [6]. To evade egg rejection by hosts, the distinct maternal lineage of cuckoo finches which parasitises prinias [7] has evolved mimicry of prinia egg patterns. Puzzlingly, however, this mimicry appears strikingly imperfect to human eyes: cuckoo finch eggs lack the scribbles of pigment which are present on all prinia eggs (figure 2*a*). Yet, prinias frequently accept eggs that lack scribbles [8]. Here, we quantify scribbles, and their contribution to the differences between host and parasitic eggs, using Minkowski functionals. These describe geometrical patterns and topological connectedness [9–11]. We also experimentally improve mimicry to test whether this influences receiver responses in prinia parents. We expected to find trait differences between prinia and cuckoo finch eggs corresponding to presence and absence of scribbles respectively. We expected little response-based differences between eggs with and without scribbles, since in nature prinias accept some eggs which lack scribbles [8].

## 2. Methods

Fieldwork was conducted on Semahwa and Musumanene Farms (ca. 16°74′S, 26°90′S) in Choma District, Zambia.

### (a) Trait quantification

We used flexible discriminant analysis (FDA; function *fda* in R package *mda* [12]) to quantify trait-based mimetic fidelity. A high-performing FDA would correctly assign eggs to species, reflecting trait differences between species.

We extracted measurements of colour and pattern traits from a representative egg (prinia *n* = 215; cuckoo finch *n* = 123) from clutches laid during 2018–2020 (alongside cuckoo finch eggs laid in 2012–2016), as described in [13]. Colour measures were extracted as in [7]; briefly, reflectance spectra were obtained using an Ocean Optics USB2000 Spectrophotometer, with a PX-2 pulsed xenon light source and an R400-7-UV/VIS reflectance probe, standardized with a Spectralon 99% White Standard (Labsphere) and a black felt cloth. Photon catches were calculated from the average of five measurements of an egg's background colour, using the R package PAVO and a blue tit *Cyanistes caeruleus* model [14]. We used two principal components of photon catches, capturing 88% of the variation, in the FDA.

Measures of principal marking size, marking size variation, pattern contrast, pattern coverage, and the extent to which pattern is dispersed between blunt and narrow poles of the egg were extracted using granularity analysis in MATLAB [8] and adaptive thresholding in the MICA toolbox in ImageJ [15], as described in [13].

Further pattern measures were quantified using NaturePatternMatch, which uses scale-invariant feature transform (SIFT) [16], to extract ‘features’ corresponding loosely to individual pattern markings. We extracted the number of SIFT features, and the mean and standard deviation of the size of the SIFT features. Two principal components, capturing 94% of the variation, were used in the FDA.

Scribbles are characterized by a large perimeter : area ratio, which the measures above cannot capture. Therefore, to quantify scribbles we deployed Minkowski functionals [9–11], which are used to characterize statistical geometries in, for example, physical patterns in reaction–diffusion equations and cosmic microwave background radiation maps. For each image, we inverted image intensity such that high intensity regions corresponded to pattern markings, applied a Gaussian filter (MATLAB function imgaussfilt) to reduce noise, and performed image thresholding with 80 thresholds (MATLAB function bwlabel) so that only regions with intensity values higher than the given threshold were retained. For each thresholded image, we computed three Minkowski measures (using the MATLAB imMinkowski toolbox [17]) which assess pattern geometry and topology: the geometrical measures of perimeter and area, and a topological measure, the Euler characteristic [17]. The perimeter P of a thresholded image is the total perimeter of all high intensity regions in it, the area A is the total area of those regions, and the Euler characteristic is the difference between the number of connected components and the number of holes in the image (i.e. a measure of pattern connectivity). To obtain a single value for each egg, we took the integral (sum Σ) of each Minkowski functional across the 80 thresholds.

**Figure 1.**
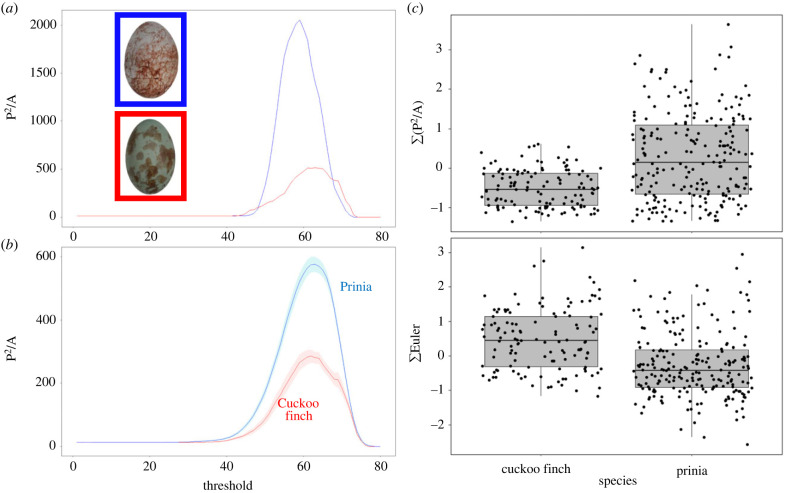
(*a*) Minkowski functionals of P^2^/A (perimeter^2^ to area ratio) across 80 thresholds for a heavily scribbled prinia egg (top egg; blue line), and a lightly scribbled prinia egg (bottom egg; red line). (*b*) Mean Minkowski functionals of P^2^/A for prinia eggs (blue) and cuckoo finch eggs (red). Shading indicates standard errors. (*c*) Boxplots showing scaled values of Σ(P^2^/A) and ΣEuler for cuckoo finches and prinias, where lower ΣEuler values indicate higher pattern connectivity.

Intuitively, the dimensionless quantity P^2^/A can capture the high perimeter and low area of scribbles (figure 1*a*). To confirm this, we tested whether painting parasitic eggs with scribbles (see below) increased Σ(P^2^/A), using a matched pairs *t*-test.

### (b) Receiver response quantification

To determine whether the presence of scribbles affected host responses, we conducted experiments (*n* = 44) largely as in [13]. Briefly, one egg from a completed host clutch was replaced with an ‘experimental' egg, with the nest observed for 4 days to determine whether the replacement egg was rejected [8,13]. Hosts were given close colour matches to human vision (cf. [13]).

**Figure 2.**
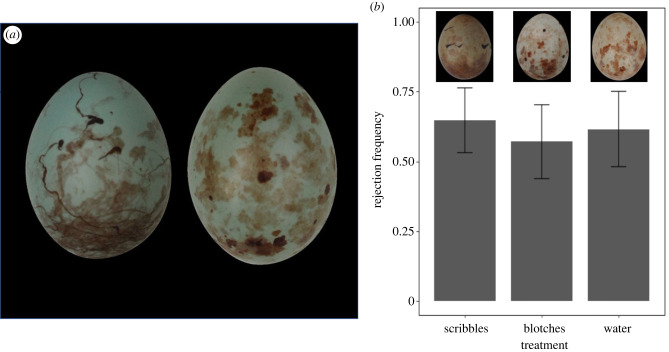
(*a*) A prinia (left) and cuckoo finch egg, illustrating presence and absence of scribbles respectively. (*b*) Rejection frequencies and standard errors for cuckoo finch eggs painted with scribbles (65%), blotches (57%) or water (62%), with examples of each treatment inset above the corresponding bars.

Unlike [13], real parasitic eggs (taken from other prinia nests) rather than conspecific eggs were used as experimental eggs. The test treatment (*n* = 17) was five scribbles painted on a real cuckoo finch egg; at least a part of one scribble could be seen by viewing any angle of the egg. Control treatments were: (a) 10 blotches totalling approximately the same area (electronic supplementary material) as the five scribbles in the test treatment (*n* = 14); or (b) five scribbles painted with water (*n* = 13) (figure 2*b*). Control (a) assessed whether the addition of paint influenced rejection. Control (b) determined a baseline rate of rejection and controlled for effects of handling the egg. Treatments were allocated randomly. We painted eggs with Standard Series Acrylic Van Dyke Brown paint (Amsterdam, The Netherlands), using a firm toothbrush bristle held in tweezers with putty. Paint colour was similar to natural pattern coloration (mean JND = 1.37; electronic supplementary material).

We analysed whether treatment affected host response (i.e. egg rejection) using logistic regression (function glm in R [18]).

## 3. Results

### (a) Trait quantification

A FDA based on colour and pattern traits correctly assigned 86% of cuckoo finch eggs and 91% of prinia eggs to species. The most important traits contributing to assignment were Σ(P^2^/A) (coefficient = 50%) and ΣEuler (coefficient = 36%; figure 1*b,c*; electronic supplementary material, table S1). Coefficients of traits indicate their contribution to discriminating between eggs.

Cuckoo finch eggs after painting with scribbles (*n* = 16; some of these were used in receiver response quantification) exhibited higher Σ(P^2^/A) values than before painting (*t*_15_ = 2.25, *p* = 0.04), although not higher ΣEuler values (*t*_15_ = 0.60, *p* = 0.56), indicating that Σ(P^2^/A) does indeed capture scribbles.

### (b) Receiver response quantification

In a model of rejection∼treatment, rejection was not predicted by treatment (treatment-scribbles: estimate ± s.e. = 0.32 ± 0.74, Z_41_ = 0.43, *p* = 0.67; treatment-blotches: estimate ± s.e. = 0.18 ± 0.79, Z_41_ = 0.23, *p* = 0.82; figure 2). Thus, the addition of scribbles to a cuckoo finch egg did not reduce its probability of rejection. In three trials, a prinia rejected one of its own eggs alongside the cuckoo finch egg; in one trial, the prinia rejected one of its own eggs while accepting the cuckoo finch egg.

## 4. Discussion

In this study, we quantified trait-based and response-based measures of mimetic fidelity in a brood parasite–host system. We showed that scribbles are captured by Minkowski functionals and result in quantifiable trait differences between hosts and parasites (i.e. imperfect mimicry). However, the addition of scribble markings to cuckoo finch eggs did not reduce the probability of egg rejection by hosts, implying that the absence of scribbles on parasitic eggs does not affect response-based mimetic fidelity.

A discriminant analysis performed well at assigning eggs to species, illustrating that consistent differences exist between host and parasitic eggs. The principal traits contributing to species assignment were the scaled ratio of perimeter to area Σ(P^2^/A) and the Euler characteristic ΣEuler.

Intuitively, Σ(P^2^/A) corresponds loosely to marking size: high values correspond to small markings or those with more perimeter such as line-like markings. Previous work [8] has shown that a coarse-grained measure of marking size (principal marking size, calculated with granularity analyses) differs significantly between prinias and cuckoo finches, with hosts exhibiting smaller marking sizes than parasites [8]. However, this measure cannot fully capture scribbles, because scribbles are characterized by both large and small size (since they are both long and thin). P^2^/A, by contrast, captures this property of scribbles. The Minkowski measure P^2^/A could therefore be applied to the wide range of biological contexts in which patterns consist of lines, such as stripes and waves [19].

Prinia eggs exhibited lower values of ΣEuler than cuckoo finch eggs. ΣEuler is a topological measure corresponding to the number of separate markings in a pattern, i.e. pattern connectivity. This result was surprising since another measure of number of markings (the number of SIFT features, extracted using NaturePatternMatch [16]), shows the opposite relationship: prinia eggs have more SIFT features than cuckoo finch eggs [13]. This apparent discrepancy may also be due to scribbles: one scribble often corresponds to multiple SIFT features, due to large changes in orientation of some SIFT features. By contrast, ΣEuler measures connectivity (low ΣEuler indicates high connectivity) and scribbles often connect multiple markings together. The addition of scribbles did not affect ΣEuler values for cuckoo finch eggs, perhaps because five scribbles were too few to significantly increase connectivity. Nevertheless, Minkowski functionals provide qualitatively different information to previously studied measures such as principal marking size and number of SIFT features.

Moving from trait-based to response-based measures of mimetic fidelity, few studies have tested whether attempts to artificially improve mimicry result in improved receiver performance (but see [20,21]). Here, we improved trait-based mimicry by painting scribbles on cuckoo finch eggs. Rejection rates across treatments were high (57–65%) despite hosts being given close colour matches; by comparison, simulations of parasitism in this system estimated that 50% of randomly laid parasitic eggs would be rejected by both prinias and another host, *Cisticola erythrops* [8,22]. This suggests that there are unmeasured traits important in rejection, leading to rates being underestimated by simulations.

The addition of scribbles to cuckoo finch eggs did not affect receiver performance, indicating that prinias do not use this trait in rejection, unlike other traits which differ consistently between parasitic eggs and their own [8,13]. Perhaps prinias are unable to perceive thin markings, due to low acuity or contrast sensitivity. While recent work has focused on acuity limits in spatial vision [23], little research has applied contrast sensitivity functions (CSFs) to spatial visual modelling. Avian CSFs are typically 10 times lower than those of humans [24], highlighting that patterns visible to humans may be invisible to birds. Thus, cuckoo finches may lack scribbles because prinias cannot use the absence of scribbles in decision-making; i.e. the ‘eye-of-the-beholder' hypothesis for imperfect mimicry [25].

Given that scribbles are not used in rejection, why are prinia eggs scribbled? Perhaps scribbles have a function unrelated to parasitism, such as egg camouflage, which predicts egg survival in other systems [26]. A close relative of *P. subflava*, *P. maculosa*, which is not parasitized by cuckoo finches, sometimes has scribbled eggs [27,28]. The function of scribbles, in prinias and in a wide taxonomic range of birds [27], merits further study.

In summary, we conducted trait-based and receiver-based analyses of mimetic fidelity in a brood parasite–host system. While discriminant analysis performed well at assigning eggs to species, prinias did not use the most salient trait—the presence/absence of scribbles on eggs—in rejection. This suggests that cuckoo finches are under relaxed selection to mimic this trait. We also introduced a novel method for quantifying patterning using Minkowski functionals, which can distinguish long, thin markings from other patterns. Beyond the specific example of egg pattern discrimination, statistical–geometrical and statistical–topological measures such as Minkowski functionals might be used to quantify markings in a wide range of biological contexts [19].

## Data Availability

Data, R code, and a description of the dataset associated with this manuscript are available from the Dryad Digital Repository: https://doi.org/10.5061/dryad.63xsj3v60 [29]. Supplementary methods and results are provided in the electronic supplementary material [30].
